# Domestication effects on social information transfer in chickens

**DOI:** 10.1007/s10071-022-01628-2

**Published:** 2022-05-04

**Authors:** Austeja Rutkauskaite, Per Jensen

**Affiliations:** IFM Biology, Linköping University, 581 83 Linköping, Sweden

**Keywords:** Domestication, Problem-solving, Stimulus enhancement

## Abstract

Red junglefowl (RJF), ancestor of all domesticated chickens, is a highly social, omnivorous bird species, presumably with a capacity for social information sharing. During domestication, birds have been selected to live in large, dynamic groups, and this may have affected their social cognition. Here, we studied social information transfer in female RJF and domesticated White Leghorn (WL) chickens. Individuals were trained to open a puzzle-box feeder by pecking a lid and we then recorded the behaviour towards the same puzzle-box feeder for birds that had either observed the trained individual (“guided”) or saw the puzzle-box feeder for the first time (“naïve”). WL were considerably faster in approaching the feeder regardless of prior demonstration and pecked more at it. Both breeds were significantly faster to approach the puzzle-box feeder and pecked more after prior demonstration, but the effects were significantly stronger in WL. We conclude that both RJF and WL can utilize social information to address a novel problem, but during domestication this ability appears to have increased. The effects can be an effect of either social learning or stimulus enhancement. Some caution in this conclusion is necessary since we tested relatively few WL. Furthermore, possible confounding explanations include higher fearfulness in RJF and different effects of dominance interactions between demonstrators and observers.

**Supplementary Information:**

The online version contains supplementary material available at 10.1007/s10071-022-01628-2.

## Introduction

Red junglefowl (*Gallus gallus*), ancestors of all domesticated chickens (Tixier-Boichard et al. 2011), are highly social and omnivorous birds, living in life-long family groups confined to small territories in the rainforests of South-East Asia (Collias and Collias 1996). Species with this type of social ecology usually show capacity for complex collaboration and social information sharing. Such sharing includes social learning (defined as the ability to copy a goal-directed behaviour after observing another individual) as well as local and stimulus enhancement, in which the general interest of another individual in a particular site or object facilitates learning about that particular item (Hecht et al. 2012). Although no systematic studies exist of this in Red junglefowl, domesticated chickens are able to obtain information from conspecifics and act accordingly, for example, by avoiding unpleasant stimuli after observing another individual experiencing it (Johnston et al. 1998) and discriminating rewarding from non-rewarding key-pecks after seeing a demonstrator pecking the correct key (Nicol and Pope 1992). Furthermore, Laker et al. (2021) showed that a novel foraging task could spread through social learning in a group of domesticated chickens. Chickens were domesticated about 9000 years ago from the Red junglefowl (Tixier-Boichard et al. 2011). Domestication has caused a gradual adaptation to life among humans, involving, e.g., reduced fearfulness and increased social tolerance (Jensen 2014). Whereas modern chickens possess a similar social behaviour repertoire as their ancestors, little is known about how social cognition has been affected by domestication (Väisänen et al. 2005; Eklund and Jensen 2011). Since domestication is associated with, e.g., reduced intra-specific competition over food and more dynamic social groupings, where group composition is usually larger than in nature and also frequently changed by adding and removing birds, we can assume that certain cognitive adaptations have occurred in relation to social interactions (Price 2002; Garnham and Løvlie 2018). For example, we can hypothesise that the ability to recognize other individuals and use social information has increased. The latter may be related to the fact that brain size and composition have been altered in domesticated birds. While total brain mass relative to body mass has been reduced, some parts, primarily the cerebellum, in fact, increased in relative size (Henriksen et al. 2016), the latter mainly through increase and enlargement of granule cells (Racicot et al. 2021). For a long time, the cerebellum has been considered to mainly control motor activity, but later research has shown its much broader involvement in social cognition and memory in many species (Van Overwalle 2009). For example, in rats, the ability to learn new actions by observing others performing the same activities relies on cerebellar involvement (Leggio et al. 2000). Most studies have so far been carried out in mammals, for example, humans and other primates, where cognitive development as well as social interactions are affected by the cerebellum (Wang et al. 2014; Adamaszek et al. 2017). In chickens, increased relative cerebellum size has been shown to improve habituating to and memorizing fearful stimuli (Katajamaa et al. 2021). It is therefore likely that there is neurobiological foundation for any modifications in cognitive abilities during domestication. Based on what we know about domestication effects on sociality and brain composition, there are thus both functional and neurobiological reasons for hypothesising that domestication may have altered the ability of chickens to process social information. To study this, we compared how prior social demonstration affected the ability to approach and solve a puzzle-box problem in both ancestral Red junglefowl and domesticated laying hens. We hypothesised that prior demonstration would increase the efficiency of problem-solving in both breeds, but that the effects would be stronger in the domesticates based on their increased cerebellum size and their domestication-induced reduction in fearfulness combined with increased social tolerance.

## Materials and methods

The complete data set is available as electronic supplementary material (table S1).

### Animals

We studied 12 female white leghorn (WL) laying hens and 41 female red junglefowl (RJF) hatched and kept in the experimental chicken facilities of Linköping University. The WL were from an outbred line (“SLU13”) originating from a Swedish breeding experiment and the RJF were from a population originating from a Swedish zoo that in turn had obtained them from Thailand. In addition, one female of each breed, taken from the same social groups as the experimental birds, was used as demonstrators. Due to limitations in the availability of the outbred WL strain, the experiment was based on relatively few birds from this breed, which should of course be kept in mind when interpreting the results. Following observations in the home-pen, we selected demonstrators that were socially dominant, as judged by their social interactions. This was done since it is known that chickens usually copy behaviour more readily from dominant individuals (Nicol and Pope 1992). Within each breed, all birds were well known to each other. All birds were of similar age (15 months at the start of the experiment and 19 months in the end) and had been kept in similar pens with the same feed from hatch until the start of the experiments. A full description of the background of the birds has been published previously (Ericsson and Jensen 2016). All chicks were hatched in small incubators and were kept in rearing pens measuring 1 × 4 m until 5 weeks old, at which point they were moved from the hatchery to the breeding facility. Here they were housed in modified aviary pens measuring 3 × 3 × 3 m with access to an outside pen of the same size. The birds had free access to commercial chicken feed, water, nest boxes and perches and platforms on different levels in the pens. They were kept in mixed-breed groups separated by sex. The indoor part of the enclosure had a light-dark cycle of 12 h, and the temperature was maintained at around 20 °C. During four weeks prior to testing, the birds received canned sweet corn (maize) daily in their home pens to make them familiar with this attractive food.

### Problem device

**Fig. 1:**
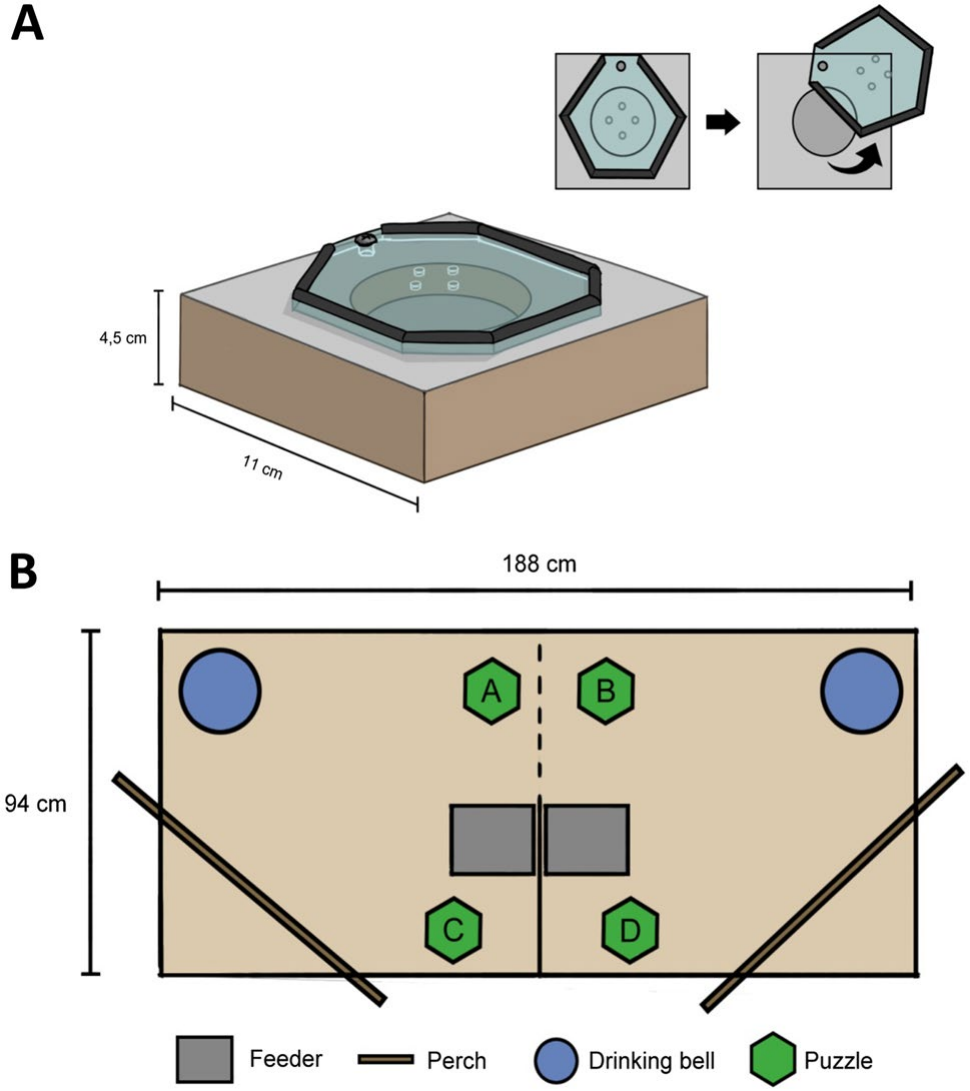
**A** The puzzle-box feeder used for the experiment. To access the food (sweet corn) in the well, the bird had to peck at the edge of the lid so it would slide open. **B** The experimental pen arrangement. Positions **A** and **B** show where the puzzle-box was placed during a demonstration trial, and **C** and **D** show the positions during the non-demonstration trials. The dotted line represents the transparent wire mesh wall, while a solid line shows a non-transparent partitioning.

The problem device (“puzzle-box feeder”) consisted of a wooden block with a circular well in the middle, covered by a transparent perforated Plexiglass lid (Fig. 1A). The well was baited with corn (highly attractive to chickens) and to access the corn, a chicken had to slide the lid to the side by means of pecking it sideways by its edge until sufficiently open. During four weeks prior to the experiment, the birds were given daily exposure in their home pens to similar feeders without lids to get fully acquainted with corn as well as the device.

### Training of demonstrators

The demonstrator birds were trained for one week to reliably open the lid. The puzzle apparatus was presented in stages of increasing difficulty. In the first sessions, it was placed with the bird with the lid fully or partially open. During the first three days of training, the birds were given 10–24 trials per day and as training continued, this was gradually increased to an average of 42 trials per day. As training progressed, the lid was gradually closed more and more until presented fully closed. The training criterion was set to a successful opening of the fully closed lid 10 times in a row. At the end of the training period, both the RJF and the WL demonstrator opened the lid consistently within 30 s after presentation for at least 10 consecutive trials.

### Experimental procedure

The tests were carried out in a separate room, in an arena consisting of two compartments (0.94 × 0.94 m each) where half the intersecting wall consisted of wire mesh allowing full view and social contact to the other side, and the other half consisted of non-transparent cardboard (Fig. 1B). Each compartment had a separate standard chicken feeder with standard chicken feed, and water ad lib. Hence, all birds had continuous access to feed and water throughout the tests. Half the tests were done with one trained demonstrator in one compartment and an observer (“guided”) in the other, and the other half with one unguided (“naïve”) bird in each compartment. Demonstrators would stay in the pen for the length of the experiment, while guided and naïve birds were left to habituate for 23 h before onset of testing. A demonstrator test (“guided”) started by placing the puzzle box feeder filled with 5–6 pieces of sweet corn together with the trained bird, within 10 cm of the wire-mesh partition to allow the bird on the other side full sight of it. After the trained demonstrator had opened the lid and consumed the corn, it was refilled and immediately placed back with the demonstrator. In total five repeated demonstrations were given. After that, the puzzle box feeder was again baited, and then placed in the compartment of the observer (guided) bird. Each test continued for 30 min and was recorded on video without any human being present in the test room. For non-demonstrator tests (“naïve”), two untrained birds at a time were placed in each of the compartments and left to habituate for 23 h. A puzzle boxfeeder was then offered to one of the birds for 30 min, this time placed next to the cardboard wall so the other bird could not see it. Following this, the puzzle boxfeeder was offered to the other untrained bird, again behind the cardboard wall. Recording was again done on video with no human presence. Trained birds will hereafter be referred to as “demonstrators”, and the untrained as “guided” or “naïve” respectively.

### Recordings

From the videos, we recorded the latency until the chicken approached the puzzle-box feeder with the head within 5 cm of it, and the total number of pecks directed at the puzzle-box feeder during the test (pecks with the beak that touched the puzzle-box feeder or the lid)). Furthermore, we recorded whether each guided and naïve bird opened the lid to uncover at least 25% of the opening during the test time. This size of the opening was judged to be sufficient for the birds to reach the corn in the puzzle-box feeder.

### Statistics

The statistical analysis was done with SPSS v. 21. We first examined whether approach latencies were affected by prior demonstration in each of the two breeds. A relatively large number of birds (mainly among the naïve Red Junglefowl) never approached the puzzle-box feeder, and they were therefore assigned a maximum approach latency of 1800 s. Since so many latencies were truncated, we then used Kaplan–Meier survival curves with Mantel–Cox Log Rank test to statistically examine the differences between breeds and demonstrator conditions (guided vs naïve), including breed and prior demonstration as the main effects in the model. For the data on the number of pecks, which were not truncated, we used Generalized Linear Models with a negative binomial distribution and log link function for testing differences. This model included the main effects of breed and prior demonstration, as well as the interaction between them. To test differences in the proportion of birds that managed to open the lid (> 25% open), we used *χ*^2^ test.

## Results

**Fig. 2:**
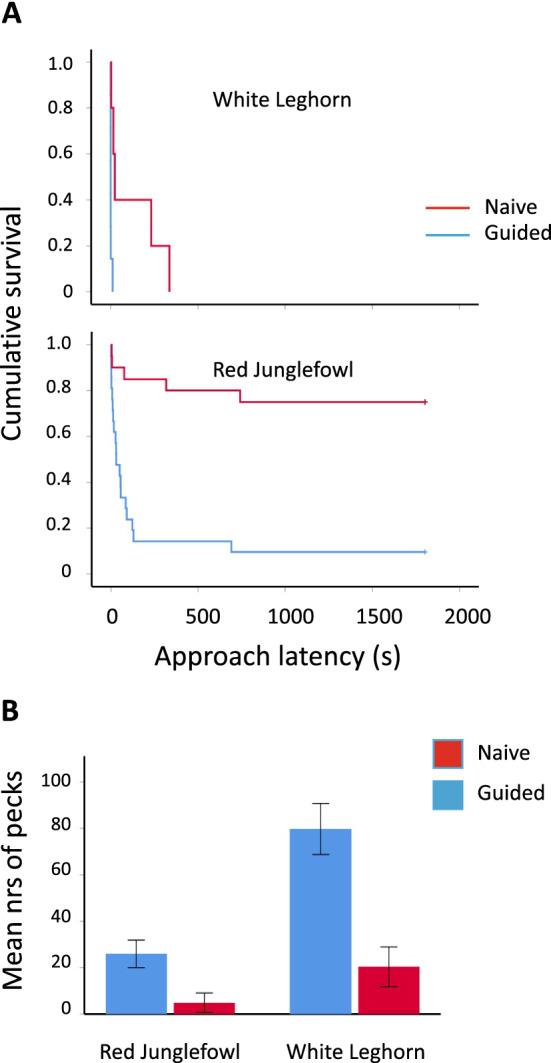
**A** Kaplan–Meier survival curves for the latencies to approach the head within 5 cm of the puzzle-box, for naïve and guided birds of domesticated white leghorns and ancestral red junglefowl. **B** Mean numbers of pecks (± SEM) directed at the puzzle-box feeder during naïve and guided trial sessions for domesticated white leghorns and ancestral red junglefowl

WL birds approached the puzzle-box feeder faster than RJF regardless of prior demonstration (Fig. 2A) (Guided: *χ*^2^ = 16.1, *P* < 0.001; Naïve: *χ*^2^ = 13.8, *P* < 0.001). In both breeds, prior demonstration significantly shortened the time to approach the puzzle-box feeder, and this effect was considerably stronger in WL than in RJF (Fig. 2A). The estimated mean latency (EML) was 50.7 times shorter for guided compared to naïve WL, while for RJF, it was only 5.9 times shorter (EML ± SEM for guided vs naïve birds; RJF: 238.8 ± 114.8 s vs 1407 ± 155.4 s, *χ*^2^ = 20.8, *P* < 0.001; WL: 2.4 ± 1.5 s vs 121.8 ± 67.8; *χ*^2^ = 8.8, *P* = 0.003). Out of 20 naïve RJF, 15 never approached the puzzle-box feeder, and 2 out of 21 guided RJF also failed to approach it, while all WL, regardless of prior demonstration, approached the puzzle-box within 335 s. WL also pecked significantly more at the lid, and in both breeds prior to demonstration significantly increased the number of pecks (Fig. 2B). The effects of both breed and demonstration were highly significant (Breed: Wald *χ*^2^ = 13.9, *P* < 0.001; Demonstration: Wald *χ*^2^ = 19.6, *P* < 0.001), and there was no significant interaction between breed and demonstration (Wald *χ*^2^ = 0.18, *P* = 0.67). A larger proportion of guided than naïve RJF managed to open the lid: 8 out of 21 guided (38%) and 2 out of 20 naïve (10%) (*χ*^2^ Likelihood ratio = 4.6, *P* = 0.031). Also, in WL more guided than naïve birds opened the lid: 5 out of 7 guided (71%) and 2 out of 5 naïve (20%). Despite the larger percentage of successful guided WL, this was not significant (*χ*^2^ Likelihood ratio = 1.2, *P* = 0.27). Only a total of five birds actually consumed the corn in the puzzle-box feeder. These were two guided RJF, two guided WL and one naïve WL.

## Discussion

We found that both domesticated White Leghorn chickens (WL) and ancestral Red junglefowl (RJF) were able to use social information to address a novel puzzle-box feeder problem. Following prior demonstration, they approached the puzzle-box feeder faster and pecked more at it. WL were generally more interested in the puzzle-box feeder, regardless of whether they had previously observed a demonstrator opening it, but prior demonstration significantly decreased the latency to approach it and increased the pecking rate towards it. The results suggest that domestication may have increased the ability to utilise social information. However, the small number of WL tested call for some caution in the conclusions, and some alternative interpretations are discussed below. It is possible that the findings are a result of social learning in chickens, where the birds observed the demonstrator solving a specific problem and then copied the behaviour when later facing the same problem (although alternative explanations are considered below). The ability of domesticated chickens to learn from conspecifics in a social setting has been demonstrated previously. For example, after observing another bird pecking an operant key, chickens are more likely to copy that behaviour when given the opportunity (Nicol and Pope 1992), and having seen another chick tasting a bitter bead, they are less likely to peck at that bead afterwards (Johnston et al. 1998). Furthermore, in a social group of chickens, novel foraging behaviour can spread by social transmission (Laker et al. 2021). In the present study, the birds had to observe experienced birds performing a particular novel behaviour, faced with a previously unknown problem. Although only a fraction of the tested birds actually managed to fully solve the problem and open the lid within the allocated test time, and an even smaller fraction actually consumed the corn, they clearly used some social information obtained from watching the demonstrator as shown by the considerably faster approach time and the increased pecking frequency. There was a clear difference between the breeds, both in their general interest in the puzzle-box feeder and in the response to prior demonstration. Both breeds were faster at approaching following demonstration, but this effect was more pronounced in WL. Guided birds also pecked considerably more at the puzzle-box feeder, with domesticated WL pecking significantly more than RJF, possibly indicating that domestication has increased the ability of chickens to use social information. This is in line with the findings that relative size of cerebellum, a brain region important in processing social information (Barton 2012) is larger in domestic birds (Henriksen et al. 2016). A possible functional reason for this may be that chickens have been selected for thriving in larger and more dynamic social groups than their ancestors, which may have favoured birds with increased social cognition. There are of course other possible explanations for our results. The findings may have been confounded by the generally reduced fearfulness of domesticated birds. We have previously shown that RJF behave in a more fearful manner than WL in a range of behavioural tests (Campler et al. 2009) and it is possible that the reluctance of RJF to interact with the novel puzzle-box feeder could be a result of this. We did, however, make efforts to reduce any confounding effects of fear by habituating all birds to corn as well as a simplified version (without lid) of the puzzle-box feeder in their home pens. They were also given 23 h of habituation to the test arena to reduce any fear of novelty of the test situation. Furthermore, stimulus enhancement (attraction to a particular stimulus that attracts another individual, rather than copying its actions towards the stimulus (Heyes 1994)) is a possible explanation for the effects of prior demonstration. However, regardless of whether the results were caused by social learning or stimulus enhancement, domesticated WL were more efficient in using the obtained information. We selected demonstrators known to be socially dominant, and it cannot be excluded that this may have affected the results. Whereas it is known that social information in chickens is more readily transferred from dominant birds (Nicol and Pope 1992), it could also have induced some reluctance in the observers to approach the puzzle-box feeder as the dominant individual was fully visible. Possibly, social dominance could have a stronger effect on RJF and contributed to the slower approach to the puzzle-box feeder in this breed. The low number of WL that were included in the study, caused by the shortage of available animals of this unique strain (the SLU13 described in “Materials and Methods”), is an obvious limitation in our study. On the other hand, all birds were hatched and reared under identical conditions, which strengthens the results, and the breed differences were so clear that it is doubtful whether they would have been altered by including more individuals. Possibly, the higher proportion of guided WL managing to open the lid would have been statistically significant with more birds in the study. An additional limitation is that we only studied females, since males are usually more competitive and aggressive and would have been difficult to manage in the demonstration setup (Nicol 2004). In conclusion, we found that both domesticated white leghorn chickens and ancestral red junglefowl were more attracted to a novel puzzle-box feeder after having observed a trained conspecific opening it to access attractive food. The effect was considerably stronger in the White Leghorns, suggesting that chickens may have acquired increased ability to utilise social information during domestication. However, alternative explanations such as reduced fearfulness may also have contributed to the results.

## Supplementary Information

Below is the link to the electronic supplementary material.

Supplementary file1 (XLSX 11 kb)

## Data Availability

The complete dataset is available as electronic supplementary material, table S1.
